# Enhancing TSH-based congenital hypothyroidism screening using machine learning and resampling algorithms

**DOI:** 10.1186/s12911-025-03312-0

**Published:** 2025-12-22

**Authors:** Alexander De Furia, Paula Branco, Matthew Henderson

**Affiliations:** 1 School of Electrical Engineering and Computer Science, University of Ottawa, 800 King Edward Ave., Ottawa, Ontario K1N 5N6 Canada; 2 Newborn Screening Ontario, Children’s Hospital of Eastern Ontario, 415 Smyth Road, Ottawa, Ontario K1H 8M8 Canada; 3 Department of Pathology and Laboratory Medicine, University of Ottawa, 451 Smyth Road, Ottawa, Ontario K1H 8M5 Canada

**Keywords:** Machine learning, Congenital hypothyroidism, Newborn screening, Rare disease, Class imbalance, Rare event detection

## Abstract

**Purpose:**

Congenital hypothyroidism (CH) is a common cause of severe intellectual disability, affecting approximately 1 in 2,000 newborns globally. Treatable with early intervention, congenital hypothyroidism has long been a target of newborn screening programs. Current thyroid stimulating hormone (TSH) based programs suffer from low positive predictive value, resulting in unnecessary diagnostic investigations. Congenital hypothyroidism screening has proven challenging for machine learning previously due to massive class imbalance and having a single well known predictor, preventing acceptable screening sensitivity. This study represents the most comprehensive evaluation of machine learning for congenital hypothyroidism screening to date.

**Methods:**

Analyzing data from 616,910 infants screened by Newborn Screening Ontario between 2019 and 2024. 12 classification and 12 resampling algorithms were trained using 4 different optimization metrics, for a total of 576 distinct models evaluated using stratified 5-fold cross-validation to ensure robustness. Models were optimized for sensitivity and then positive predictive value using various metrics. Model explainability was assessed using SHAP values and feature importances.

**Results:**

We were able to create a model achieving 16.8% PPV while maintaining 100% sensitivity using a RUSBoost classifier and Gaussian Noise resampling. This represents a 60% improvement in positive predictive value over the current approach. TSH remained the dominant predictor as in current screening, but our model was able to include minor amounts of additional information from other features to improve performance.

**Conclusion:**

These machine learning algorithms show no missed cases of CH and are able to significantly improve performance across robust testing. The findings suggest that machine learning offers a promising avenue for refining TSH-based CH screening processes, reducing false positives, and alleviating unnecessary stress and costs associated with current methods used by the majority of newborn screening programs globally.

**Supplementary Information:**

The online version contains supplementary material available at 10.1186/s12911-025-03312-0.

## Background

### Congenital hypothyroidism

Congenital hypothyroidism (CH) is a condition characterized by a dysfunction of the thyroid gland present at birth. It is one of the most common causes of intellectual disability, affecting approximately 1 in 1,400 to 2,800 newborns globally [1]. The thyroid gland, located in the lower neck, plays a crucial role in the regulation of metabolism, growth, and development. CH can result from various etiologies, including thyroid dysgenesis, dyshormonogenesis, and central hypothyroidism, each of which can lead to irreparable mental and physical retardation [2]. Early detection and treatment of CH are essential and highly effective in preventing long-term complications and ensure optimal neurodevelopmental outcomes [3].

Newborn Screening (NBS) programs have been instrumental in the early detection of CH, enabling timely intervention and preventing adverse outcomes [3]. The primary screening test for CH involves measuring Thyroid-stimulating hormone (TSH) levels in Dried Blood Spots (DBS) collected from newborns [4]. Elevated TSH levels above a program-specific threshold indicate a screen-positive result, prompting further diagnostic evaluation [4]. While TSH-based screening programs have been successful in detecting CH, they are associated with high false-positive rates, leading to unnecessary parental anxiety, repeat testing, and healthcare costs [4].

Generally characterized by insufficient thyroid hormone production, the specific etiologies of CH vary in the resulting endocrine profile, with some cases presenting elevated TSH levels and others with Thyroxine (T4) [2]. TSH based screening methods will more likely miss central hypothyroidism cases, where TSH levels can be normal, but T4 levels are low [2]. T4 based screening methods will be more likely to miss cases of dyshormonogenesis, where T4 levels are normal, but TSH levels are elevated [2]. The Dutch newborn screening program has successfully integrated TSH, T4, and Thyroxine-binding globulin (TBG) measurements into their screening algorithm, significantly improving the Positive Predictive Value (PPV) of their program across CH variants [5]. Given the rarity of central CH (1 in 13,000 to 25,000 live births) compared to primary CH (1 in 3,000 to 4,000 live births), the majority of screening programs worldwide rely on TSH measurements for screening [1, 4]. In approximately 17% to 40% of cases, CH may be transient, where T4 is reduced and TSH elevated after birth but later recovers to normal levels [6]. In this study, both central and primary CH are considered positive, but transient CH is considered negative. Treatment of transient CH is still an ongoing debate within NBS at the time of this study [6].

### Current NSO CH screening process

Newborn Screening Ontario (NSO) utilizes a multi-tier, TSH based, screening process to screen for CH. This process involves an initial TSH measurement, with a cutoff of 14mIU/L, followed by secondary measurements of the same dried blood sample if the initial measurement is above the cutoff [7]. If the average TSH of the three measurements is above 17mIU/L, the infant is referred to a specialist for further diagnostic evaluation [7]. The process is illustrated in Appendix A. The PPV of the entire process is 38.5% for all CH variants, with a PPV of 10% for the first tier of screening [7]. The current screening process has a sensitivity of 100% and a specificity of 99.1% [7]. By using multiple DBS measurements, the confidence in an elevated result is increased, reducing the number of false positives and improving the PPV of the program [7]. This process however is not perfect and can be improved upon. Our goal is to use machine learning to further improve the PPV of the current screening process while maintaining a sensitivity of 100% relative to the existing methods.

### Machine learning based screening

Machine learning has emerged as a promising tool in newborn screening programs, enhancing the accuracy and efficiency of rare disease detection [8]. Although many improvements arose from using machine learning, they suffer from two major pitfalls, the class imbalance problem and the target of 100% specificity [8–10]. These issues are particularly prevalent in CH screening programs, where the incidence rate is particularly low, and the target of 100% sensitivity is essential to prevent severe adverse outcomes [1].

Zarin Mousavi et al. [11] investigated the application of machine learning to CH screening using a dataset of 4812 cases from the Health Center of Alborz, Iran. Of the cohort, 75 cases were labelled as positive for CH, falling into both permanent and transient CH, representing an imbalance ratio of approximately 1:64 which is not representative of the reported global incidence rate [4]. The study evaluates four primary classifiers: Multilayer Perceptron (MLP), Support Vector Machine (SVM), Iterative Dichotomiser 3 (ID3), and Chi-squared Automatic Interaction Detection (CHAID). These classifiers were both individually trained and also combined with ensemble methods ADABoost and Bagging. Common approaches for handling class imbalance, such as resampling, were not employed. Accuracy was also used as a primary evaluation metric; considered bad practice within imbalanced learning, as it provides misleading results [12]. The noted results were misleading, highlighting an accuracy of 99.58% but ultimately poor performance, with a maximum recall of 73.33%, a PPV of 100%, from an SVM-Bagging model. Given a raw dataset of 194 attributes, 17 remained after preprocessing, a notable variation in approach from Stroek et al. [13] and Jansen et al. [14]. The training dataset included features such as family history of thyroid disease and delivery method, which were shown to have substantial output on predictions by SVM-Bagging attribute weights. The authors recommended further research with larger datasets, more features, and more advanced machine learning algorithms to improve the performance of CH screening programs.

Stroek et al. [13] was able to successfully demonstrate improvements using machine learning for CH within the Dutch NBS program. This study used DBS collected 72 to 168 hours post-birth, later than the 24 to 72 hours at NSO. Additionally, the study utilized multiple endocrine features including T4, TBG, TSH, T4/TBG ratio, and T4 deviation from the daily mean of all T4 measurements. The dataset is composed of 458 cases of primary CH, 82 cases of central CH, 2,332 false positives, and 1670 random healthy controls. This cohort is not representative of the expected incidence rate of CH, with an imbalance ratio of approximately 1:9. Using SMOTE and random forest in 10-fold cross validation, the study achieved an increase in PPV from 21% to 26% while maintaining 100% sensitivity relative to the current NBS program [15, 16]. The study did not compare different solutions for class imbalance, nor did it comment on the optimization method used. The study also did not comment on the generalizability of the model to the general population. The study was able to identify endocrine markers as the most important features in the model, with TSH being the most important, followed by T4 deviation from the mean, TBG, T4/TBG ratio, and T4. This seems to be reflective of the fact that primary CH was more common within the positive population. The study was able to demonstrate the potential of machine learning in CH screening, but was limited by a small dataset and a lack of comparison of different algorithms for class imbalance. Moreover, Stroek et al. [13] relies on access to more features such as T4 and TBG that are not typically available in other programs, such as the one at NSO [4].

Building upon the work of Stroek et al. [13], Jansen et al. [14] further improved the PPV of the Dutch NBS program from 26% to 46% while maintaining 100% sensitivity by integrating 21 amino acids (AA) and acyl carnitiness (AC) into a new model. Multiple of these AA,s and ACs were found to correlate to varying degrees with T4 and TBG. The specific dataset used was derived in a similar manner to the previous study, with 1079 false positive referral cases, 431 cases of primary CH, 84 cases of central CH, and 1842 true negative healthy controls. The study used SMOTE and random forest in a 67% training and 33% stratified testing split. The study did not compare different algorithms for class imbalance, nor did it comment on the optimization method used. The study found that endocrine markers were the most important features in the model, with TSH being the most important, followed by T4 deviation from the mean, TBG, T4/TBG ratio, and T4. The study was able to demonstrate some potential of machine learning in CH screening, but was limited by a small dataset (*n* = 3,436), unrealistic imbalance ratio (IR = 1:8), and a lack of comparison of different algorithms for class imbalance. It does, however, compellingly demonstrate the potential of machine learning in improving the PPV of CH screening programs.

In both of these studies by Stroek et al. [13] and Jansen et al. [14], diagnostic accuracy was calculated using Receiver Operating Characteristic (ROC) curves. Random Forest models predict per class probabilities which are classified based on a decision threshold, typically 50% [17]. By adjusting the decision threshold, one can adjust the sensitivity-specificity tradeoff [17]. The ROC curve can be informative about these tradeoffs, however, there is a risk in retroactively choosing a threshold that maximizes the performance of the model on the test set [12]. This can lead to overfitting and poor generalization to new data [12]. This is another aspect in which our study aims to improve upon the current literature, ensuring that our models are generalizable to new data without information leakage.

The class imbalance problem CH screening data suffers from is a well-known problem and an area of ongoing research in machine learning. Several special-purpose learning algorithms and resampling techniques are available to address the imbalance problem, each with its strengths and weaknesses. Resampling techniques are among the most popular and easy-to-use strategies working as out-of-the-box solutions. However, the effectiveness of different techniques seems to depend on the dataset characteristics and the underlying distribution. These methods can significantly improve the learnability of a given dataset, but the selection of the best method to apply is not an easy task requiring a comprehensive experimental evaluation. Thus, limiting our study of CH screening to a single learning algorithm or resampling technique would not provide a good understanding of the potential of machine learning in this area.

In this paper, we aim to overcome the limitations of previous studies, by leveraging a significantly larger dataset of over 600,000 cases that is more representative of the true class imbalance problem in CH screening. Moreover, we provide a thorough experimental evaluation of multiple learning algorithms using a large set of resampling techniques and different optimization metrics. Our study shows that RUSBoost, with Gaussian Noise (GN) resampling is able to improve the PPV of the initial screening at NSO from 10% to 17% maintaining the sensitivity of the current program. This study provides a comprehensive evaluation of machine learning algorithms and resampling techniques for CH screening, demonstrating the potential of machine learning in improving the performance of CH screening programs.

## Materials & methods

### Newborn screening data

This is a retrospective study of all infants screened by NSO from July 29, 2019, to March 30, 2024. The dataset was sourced from the NSO screening database and underwent preprocessing to remove incomplete or inconsistent entries. The final dataset includes 59 features and 616,910 cases. The features used in the study are listed in Table 1. This study focuses on infants with a complete panel of metabolic data, birth weight, and gestational age, and those with TSH serum levels measured within the appropriate timeframe (24 hours to 7 days post-birth). Features used during screening are standard within the NSO program and are used to screen for a variety of conditions, not just CH. The dataset includes features such as acyl-carnitines, amino acids, hemoglobin, endocrine markers, and immune markers. The dataset is highly imbalanced, with a positive to negative ratio of 1:1809, reflective of the expected occurrence rate of CH [4].

**Table 1:** Features used in this study

Category	Features
Clinical Features	Gestational Age, Birth Weight, Age at Sample Collection, Multiple Birth, Sex, Transfusion Status
Acyl-carnitines	C0, C2, C3, C3DC, C4, C4DC, C4OH, C5, C5:1, C5OH, C5DC, C6, C6DC, C8, C8:1, C10, C10:1, C12, C12:1, C14, C14:1, C14:2, C14OH, C16, C16OH, C16:1OH, C18, C18:1, C18:2, C18OH, C18:10 H
Amino Acids	Arginine, Phenylalanine, Alanine, Leucine, Ornithine, Citruline, Tyrosine, Glycine, Valine, Succinylacetone, Methionine
Hemoglobins	Adult Hemoglobin HbA (A),
	Fetal Hemoglobin HbF (F), Acetylated HbF (F1),
	HGB_Pattern, Sickle Cell Trait (FAST)
Endocrine Markers	Thyroid stimulating hormone (TSH)
	17-Hydroxyprogesterone (17-OHP)
Enzyme markers	Biotinidase (BIOT)
	Galactose-1-phosphate uridyltransferase (GALT),
Immune Markers	T cell receptor excision circles (TREQ QN)

In the development of machine learning based models for CH screening, the consistency and quality of data are crucial. Consistent data ensures that the models are trained on reliable and representative samples, which is crucial for accurate predictions. Inconsistent or incomplete data can lead to biased models that fail to generalize to new, unseen data, potentially resulting in poor performance. This involves removing entries with missing values, correcting errors, and standardizing the data collection process. Similar to the current screening method, we are only able to effectively screen infants with a complete panel of metabolic data, birth weight, and gestational age, and those with TSH serum levels measured within the appropriate timeframe (24 hours to 7 days post-birth). The timing of this measurement is crucial, as infant TSH levels spike and are expected to return to normal levels within the first few days post-birth [18]. Cases outside this timeframe are removed, as they should be remeasured to ensure accurate results.

In NBS it is standard to maintain strict requirements to determine if screening of a particular DBS is applicable, in the case that the organizations’ standards are not met, repeat sampling and/or testing are requested. In keeping with this we apply a standardized approach to preprocessing, removing cases that do not meet integrity requirements, and ensuring reproducibility and ease of use on other datasets. In the case that a future sample did not meet the standards required by our preprocessing, a repeat sample would be requested. This follows a well-defined, multistep process:

*Feature Naming* Removing special characters and ensuring consistent formatting algorithmically. No data is removed. Feature names used are listed in Table 1.

*Cleaning of values*

1. Valid regex is defined for each column.
2. Any row with values not meeting all feature regex’ are removed.
3. User defined features are mapped into categorical, boolean, or numerical values.
4. Cases outside acceptable ‘age of collection’ numerical range are removed.

*One Hot Encoding* All categorical values are one hot encoded, or if specified, binary encoded. Sex is binary encoded to account for Male, Female, Unknown, and Intersex without excess features which could lead to overfitting.

*Numerical Encoding* Numerical columns are encoded as integers if there is no floating point information present. Otherwise, all features are encoded as default NumPy floating point values.

*Label Encoding* “Diagnosis” strings are mapped into their respective binary classifications of Positive and Negative. Mappings and occurrence counts are found in Table 2.

**Table 2:** Screen positive diagnoses and labels in the NSO dataset

Diagnosis	Label	Population Size
CH Ectopic	Pos	20
CH Athyreosis	Pos	12
CH Idiopathic	Pos	280
CH Hypoplastic	Pos	2
CH Dyshormongenesis	Pos	7
CH Apparent Athyreosis	Pos	1
CH Secondary to Amiodarone	Pos	1
CH Presumed Dysgenesis or Dyshormongenesis	Pos	153
Deceased	Neg	3
DERF Pending	Neg	9
Not Affected	Neg	472
Iodine Exposure	Neg	1
Transient TSH Elevation	Neg	136
Maternal PTU/Graves Disease	Neg	19
Lost to follow up	Neg	6
Negative	Neg	674460

**Fig. 1:**
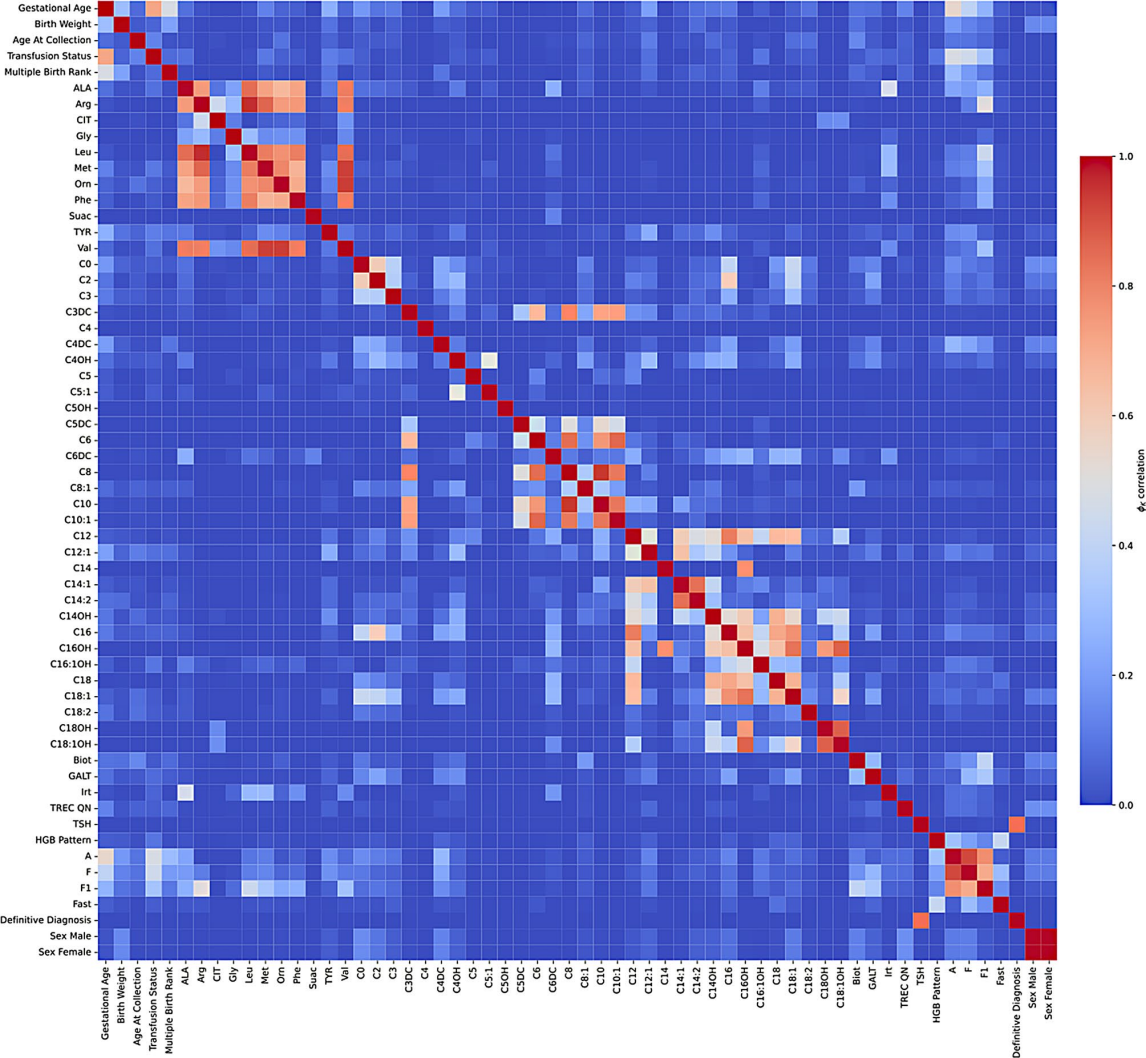
$\phi_K$Correlations between features in the NSO dataset

**Fig. 2:**
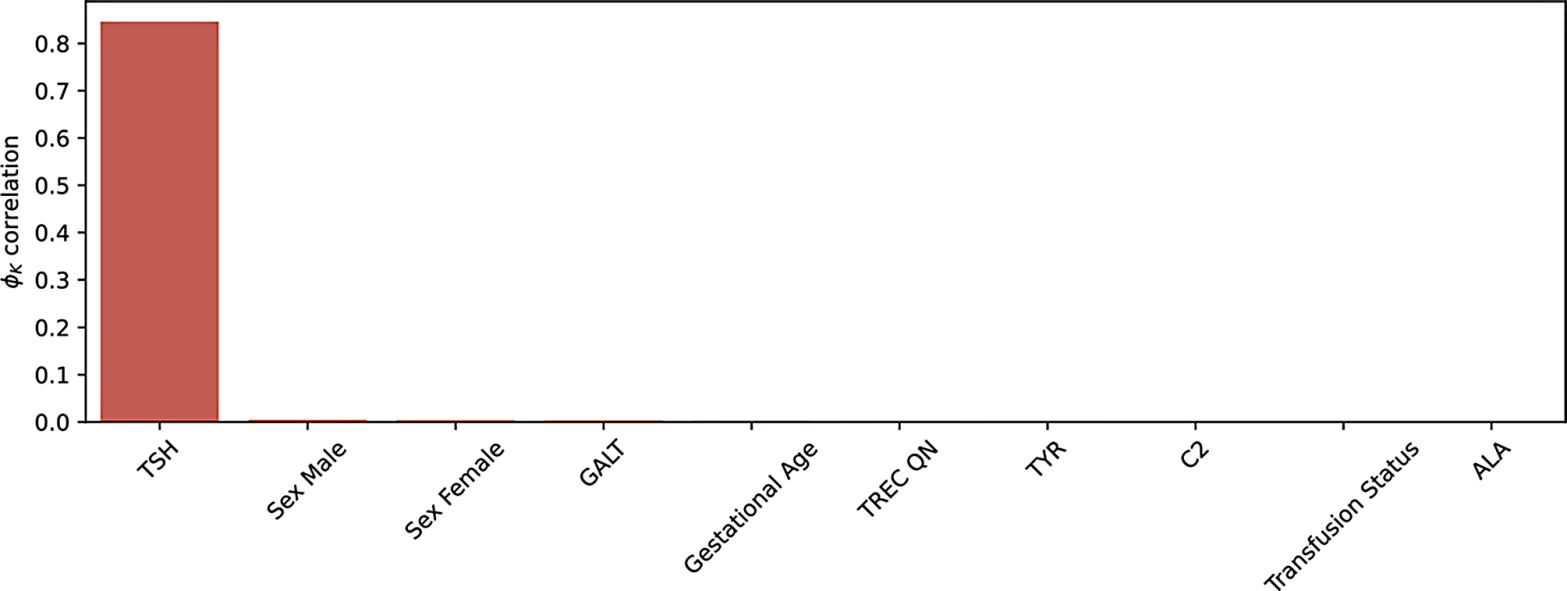
$\phi_K$Correlations between features and definitive diagnosis in the NSO dataset

**Fig. 3:**
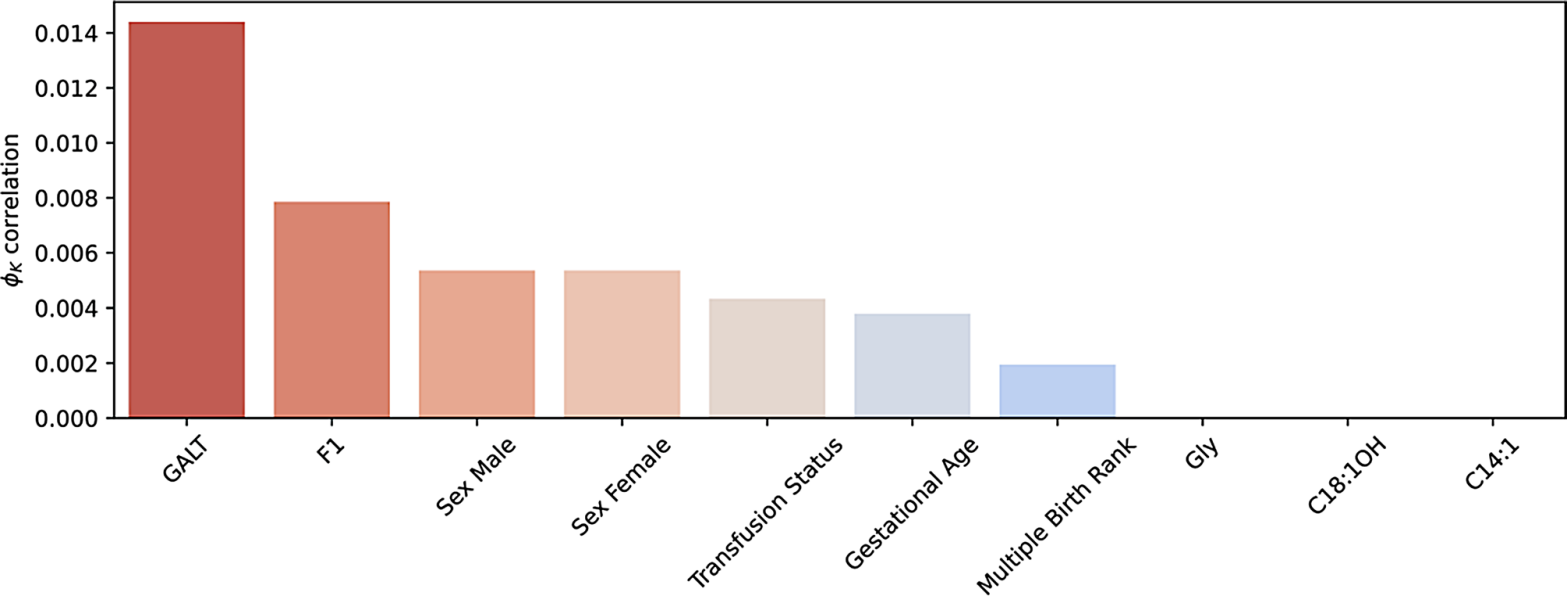
$\phi_K$Correlations between features and TSH in the NSO dataset

After the data preprocessing steps described, we conducted an exploratory data analysis. Following the work of Jansen et al. [14] $\phi_K$ correlations between the feature set and both TSH and definitive diagnosis were calculated [19]. The results of this analysis showed that all features except TSH had a negligible correlation with the definitive diagnosis. The correlation between TSH and the definitive diagnosis was 0.8, indicating a strong positive correlation. The distribution of TSH levels is a long tailed distribution in which using the current screening cutoff, of 14mIU/L, would result in 2,941 false positives and 342 true positive cases. There are two outliers in TSH concentrations with values of 15.2mIU/L and 15.4mIU/L, all other cases are above 15.9mIU/L. These two cases are likely due to measurement variability and would typically be remeasured to ensure accurate results. For our purposes, however, they will be included in the dataset as we are hoping to remove the need for remeasurement. There are clear inter-feature correlations within the dataset, shown in Fig. 1, with the majority of features having low correlations ($\le0.05$). Figure 2 shows the low feature correlations with definitive diagnosis and Fig. 3 with TSH.

### Experimental settings

#### Classifiers

We investigate a wide range of potential learning algorithms and resampling techniques, which aligns with the current understanding of the class imbalance problem [20]. The chosen set of 576 models represents the largest and most comprehensive evaluation of Machine Learning Machine Learning (ML) to CH screening. Choosing these algorithms can be a largely arbitrary process, as the best algorithm for a given problem is most often unknown [20]. This choice can have a significant impact on the performance of models, and is highly dependent on the dataset [20].

The classification algorithms chosen cover a wide variety of common classes of machine learning algorithms. The models selected are Decision Tree, Random Forest, Balanced Bagging, Balanced Random Forest, XGBoost, ADABoost, RUSBoost, and Linear SVC (outlined in Table 3). These models were selected based on their performance in other studies and their ability to handle imbalanced datasets [8, 21, 22]. Three major types of models used are decision trees, tree based ensembles (bagging and boosting), and linear separator models. Multilayer perceptron or neural network approaches were intentionally avoided in this study due to the computational expense of training these models.

**Table 3:** Classifiers used in the study. $^*$ trained with and without balanced subsampling

Classification Algorithm	Description
Decision Tree$^*$	Tree-based classifier
Random Forest$^*$	Decision Tree Ensemble
Balanced Bagging	Bagging with balanced class weights
Balanced Random Forest$^*$	Random Forest bootstrapping minority class
XGBoost	Regularized Gradient Boosting
ADABoost	Boosting model
RUSBoost	Boosting with random undersampling
Linear SVC$^*$	Linear Support Vector Classifier

Studies employing these classifier algorithms do not tend to employ balancing techniques, a key component of our study. “Balancing” refers to two concepts, balanced models and balanced class weights. The former refers to variations of algorithms with added internal resampling to balance the training examples between the classes [17]. The latter refers to the adjustment of class weights to balance the importance of each class in the model [17]. Each decision tree based model, including ensembles, was trained with and without balanced class weights, to reduce bias towards the majority class. Two major categories of ensemble models were selected, boosting and bagging. The linear and base decision tree models were included as a baseline to compare against the performance of the more complex models. The linear model was also trained with and without balanced class weights. This represents a comprehensive collection of ML algorithms for classification.

Other machine learning approaches to CH screening have included smaller sets of classifiers that we intentionally did not select to be a part of our experiment space to reduce the already high complexity, instead opting for more standard approaches within the domain of class imbalance machine learning. Zarin Mousavi et al. [11] used four different classification algorithms: MLP, ID3, SVM, and CHAID. We use SVM based classifiers, also known as Support Vector Classifier (SVC), and decision tree based methods similar to ID3 and CHAID. The MLP classifier was specifically not included due to its increased training cost, and well established limited performance on datasets with this level of class imbalance. Zarin Mousavi et al. [11] also included ensemble variants of these models that we believe overlaps well with our various selections of both Bagging and Boosting ensembles.

#### Performance assessment

Stratified 5-fold cross validation was employed to ensure that models did not overfit to the training data and were generalizing well to unseen data [22]. At each fold, the model was trained using Bayesian hyperparameter optimization, a process that treats hyperparameter tuning as a sequential optimization problem [23]. The optimization metrics used in this study are F1, F10, Recall, and FPFN; defined in Table 4. Each of these metrics was used for training and evaluation of all models. We will refer to a given metric as “scorer” when it is used during the training process, i.e., during model optimization. This means that, for instance, we can use as “scorer” the recall, and then observe the model results on F1. Depending on the metric used during training, the model receiver operating characteristics will change [12, 22]. This allows us to select a model based on our desired performance characteristics; in our case prioritizing sensitivity and then specificity.

**Table 4:** Evaluation metrics used in the study

Metric	Equation
False Positive Rate	$\frac{\text{FP}}{\text{FP} + \text{TN}}$
False Negative Rate (FNR)	$\frac{\text{FN}}{\text{FN} + \text{TP}}$
PPV	$\frac{\text{TP}}{\text{TP} + \text{FP}}$
Recall	$\frac{\text{TP}}{\text{TP} + \text{FN}}$
F1	$2 \times \frac{\text{PPV} \times \text{Recall}}{\text{PPV} + \text{Recall}}$
F10	$2 \times \frac{\text{PPV} \times \text{Recall}}{10 \times \text{PPV} + \text{Recall}}$
FPFN	${\left({\max \left\{\text{FPR}, 10^{-8} \right\} + \max \left\{\text{FNR}, 10^{-8} \right\}}\right)}^{-1}$

#### Resampling

One approach to addressing class imbalance in machine learning is to resample the training set, to create a more balanced representation of the data which is easier to learn from, rather than necessarily representing the underlying distribution. We evaluated eight resampling techniques: Random Over Sampler, Random Under Sampler, SMOTE, Adaptive Synthetic Sampling (ADASYN), Tomek Link Undersampling, Near Miss, Borderline SMOTE, and Gaussian Noise (GN), which are outlined in Table 5. Each of these techniques has its own strengths and weaknesses, and the choice of resampling technique can have a significant impact on the performance of the model [22]. Each algorithm uses the default hyperparameters from Lemaître et al. [17] with a resampling ratio of 0.02 unless otherwise specified. This can be done through several variations of undersampling the majority class or oversampling the minority class. The hyperparameters for each resampling technique were selected based on the recommendations of the imbalanced-learn library [17]. The specific values and configurations can be found in the accompanying http://GitHub repository [24]. The resampling algorithms used in the study are standard implementations from the imbalanced-learn library [17], except the Gaussian Noise (GN) strategy adapted from the “Noisy Replicate” algorithm [25]. The large dataset size was a limiting factor in selecting resampling algorithms; due to computational constraints, we were unable to evaluate popular clustering methods such as Cluster Centroid resampling or K-Means SMOTE [17].

**Table 5:** Resampling algorithms used in the study

Resampling Algorithm	Description
SMOTE	Synthetic Minority Over-sampling Technique
ADASYN	Adaptive Synthetic Sampling
Borderline SMOTE	SMOTE using borderline points
Random Under Sampling	Randomly remove majority points
Random Over Sampling	Randomly duplicate all minority points
Gaussian Noise	Random Over Sampling adding random noise
	Table 6 shows the hyperparameters used
Tomek Links	Drop majority points near minority points
Near Miss	Drop majority points far from minority points
None	No resampling is performed

Our GN implementation adjusts the “Noisy Replicate” algorithm for improved parameterization and flexibility, as outlined in Appendix Section 4. The GN resampling algorithm introduces small amounts of noise to existing points in the minority class, creating new synthetic samples that are representative of the original point. Synthetic samples are created by adding a small amount of random noise based on the mean and standard deviation of the feature. Compared to other synthetic resampling techniques like SMOTE, GN is less likely to interpolate into areas that are representative of the true distribution. This can be particularly useful in the case of CH screening, where the true distribution of the data is not known and may be multi-modal. The cost of GN resampling is that it may lower the performance of the model by introducing noise into the training set. This is a trade-off that must be considered when selecting a resampling technique. There are three parameters that are involved in our implementation: proportion of features to apply noise to, multiplier of standard deviation, and ratio of synthetic samples to real samples. Three variations of GN were evaluated in the study, each with different hyperparameters, the specific values are shown in Table 6.

**Table 6:** Hyperparameters for gaussian noise resampling

Combination	Feature Ratio	SD Multiplier	Synthetic Point Ratio
GN1	25%	0.01	2:1
GN2	25%	0.05	2:1
GN3	75%	0.05	2:1
GN4	25%	0.05	5:1

#### Search space evaluation

Given the large number of possible combinations across our search space of classifiers, resamplers, cross-validation strategies, and optimization metrics, we employed a custom runtime evaluation framework to run each combination and evaluate the performance of each model. We then manually examine the results to determine the best-performing model. This process is outlined in the Appendix, Section 2 - Search Space Evaluation. Further details can also be found in the accompanying http://GitHub repository [24].

As models are trained and tested using stratified 5-fold cross validation, all metrics in Table 4 are calculated and stored for each fold, as well as the confusion matrix values. The predictions made for each case at each fold are stored, along with predicted probabilities if supported by the given classifier. These probabilities can be used to evaluate ROC and Area Under the Curve (AUC) for later evaluation. We also use these predictions and probabilities in determining agreement between models across the data distribution, which we describe in Section 2.4.

### Explainability

The majority of algorithms used in this study create black box models, meaning that the internal mechanisms of the model are not easily interpretable. This can pose a challenge in the adoption of these models in healthcare, as the decisions are not easily interpretable by clinicians [26]. We use two methods to attempt to explain the mechanisms of the model in this study, SHapley Additive exPlanations (SHAP) values, and feature importance. While these do not provide a perfect explanation of the model, they do provide some insight into the mechanisms of the model and can be used to help determine the potential impact of feature values in predictions made by the model as a whole and on a case by case basis.

SHAP values are a powerful technique for interpreting the output of machine learning models, providing a unified measure of feature importance that is consistent across different models and can be used to explain the output of any machine learning model [27]. To calculate SHAP values, the contribution of each feature to a prediction is determined by comparing model outputs with and without that feature present, averaged over all possible feature combinations. This process assigns each feature an importance value for every individual prediction. SHAP values have several key properties that make them useful for model interpretation:

- **Local Accuracy**: They are consistent and locally accurate, meaning the sum of feature attributions equals the model output.
- **Global Accuracy**: They provide both global feature importance across the entire dataset and local explanations for individual predictions.
- **Feature Interaction**: They capture feature interactions, allowing for the identification of complex relationships between features.

We use SHAP values in a bee swarm plot to summarize the importance of features in the model. This plot shows the distribution of SHAP values for each feature, allowing us to identify the most important features in the model and their impact on predictions based on the value.

Raw feature importances can also be useful in understanding the mechanisms of the model. Feature importances are calculated by the model during training and provide a measure of the importance of each feature in the model. These importances are calculated based on the reduction in impurity that each feature provides when used in a decision tree. This is a slightly more straightforward approach to explainability than SHAP values, but can still provide valuable insight into the mechanisms of the model despite not explaining any given case. Other methods of explainability may yield more insight into the model but are not used in this study as they are not as widely used or understood as SHAP values and feature importances [28].

### Combinations of models

It is possible and often expected that different models may learn to represent different aspects of the data distribution to varying degrees of success. This is the motivation behind ensemble models which for instance combine many decision trees into a random forest. This strategy can leverage the strengths of each model to create a more robust and accurate combined model. This is particularly useful in the case of imbalanced datasets, where different models may be more or less effective at learning. In this study, we evaluate the performance of different combinations of classifiers and resamplers to determine the best-performing model.

We investigate unanimous voting, where each model votes on the class of a given sample; if all models agree on a positive prediction, the sample is predicted as positive. By optimizing first for recall, we can ensure that the model is not biased towards the majority class, and then improve the PPV by combining models in this way. There are alternative methods of combining models such as weighted voting which involves heuristically tuning the contributions of different models; this may be interesting to explore in future work. We evaluate these combinations of models after having trained the constituent models individually in the same manner as the rest of the study.

This tool is largely a mechanism to determine the similarity of models. If there is a large change in performance by combining them, it is likely that the models are learning different aspects of the data distribution. The converse is also true. This can be useful in determining the generalizability of the model to the general population, but further testing and validation would be required.

## Results

**Fig. 4:**
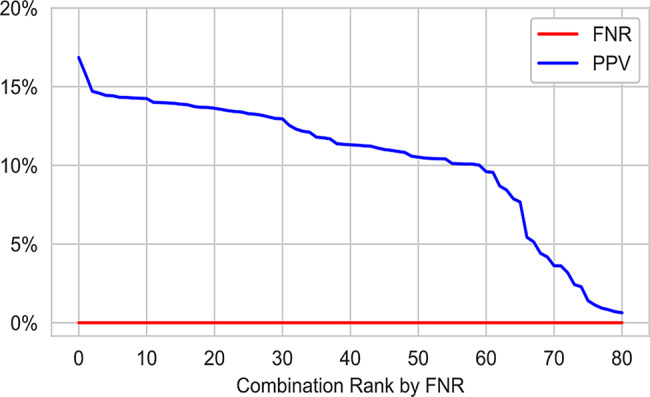
Key performance characteristics of acceptable sensitivity models in the search space

We identified 81 individual models from our search space of 576 models that achieved a 100% sensitivity across all 5 folds of stratified cross-validation. The performance metrics for the top 10 combinations sorted by PPV are summarized in Table 7. majority of the 81 models with acceptable sensitivity were not able to achieve a PPV above 10%, shown in Fig. 4. The best performing model, RUSBoost with FPFN optimization and Gaussian Noise resampling, achieved a PPV of 16.8% and a sensitivity of 100% across 5 folds of stratified cross-validation. This represents a 60% improvement over the current first tier of screening with a PPV of 10.5%.

**Table 7:** Top 10 results with 100% sensitivity, sorted by precision (PPV) using the original dataset

Classification Algorithm	Scorer	Resampling	FN	PPV$_\mu$	PPV$_\sigma$
RUSBoost	FPFN	GN$\left(25\%, 1\%, 2\right)$	0.0	0.168	0.028
Balanced Bagging	F10	GN$\left(25\%, 1\%, 2\right)$	0.0	0.158	0.012
Balanced Bagging	F10	GN$\left(25\%, 5\%, 5\right)$	0.0	0.147	0.010
RUSBoost	F10	Near Miss	0.0	0.146	0.014
RUSBoost	F10	Tomek Links	0.0	0.144	0.016
RUSBoost	FPFN	Near Miss	0.0	0.144	0.016
Balanced Bagging	F1	Tomek Links	0.0	0.143	0.012
Balanced Random Forest	FPFN	GN$\left(25\%, 1\%, 2\right)$	0.0	0.143	0.011
RUSBoost	Recall	Near Miss	0.0	0.143	0.016
RUSBoost	Recall	Tomek Links	0.0	0.143	0.011

These GN hyperparameters reflect a relatively small shift in Euclidean space from the original points. This model does have a slightly elevated PPV standard deviation compared to the other models of 2.8%. RUSBoost and GN dominate the remainder of the search space, with 60% of the top models using RUSBoost and 40% using a variation of GN. These combinations achieve very similar performance despite varying internal approaches.

**Fig. 5:**
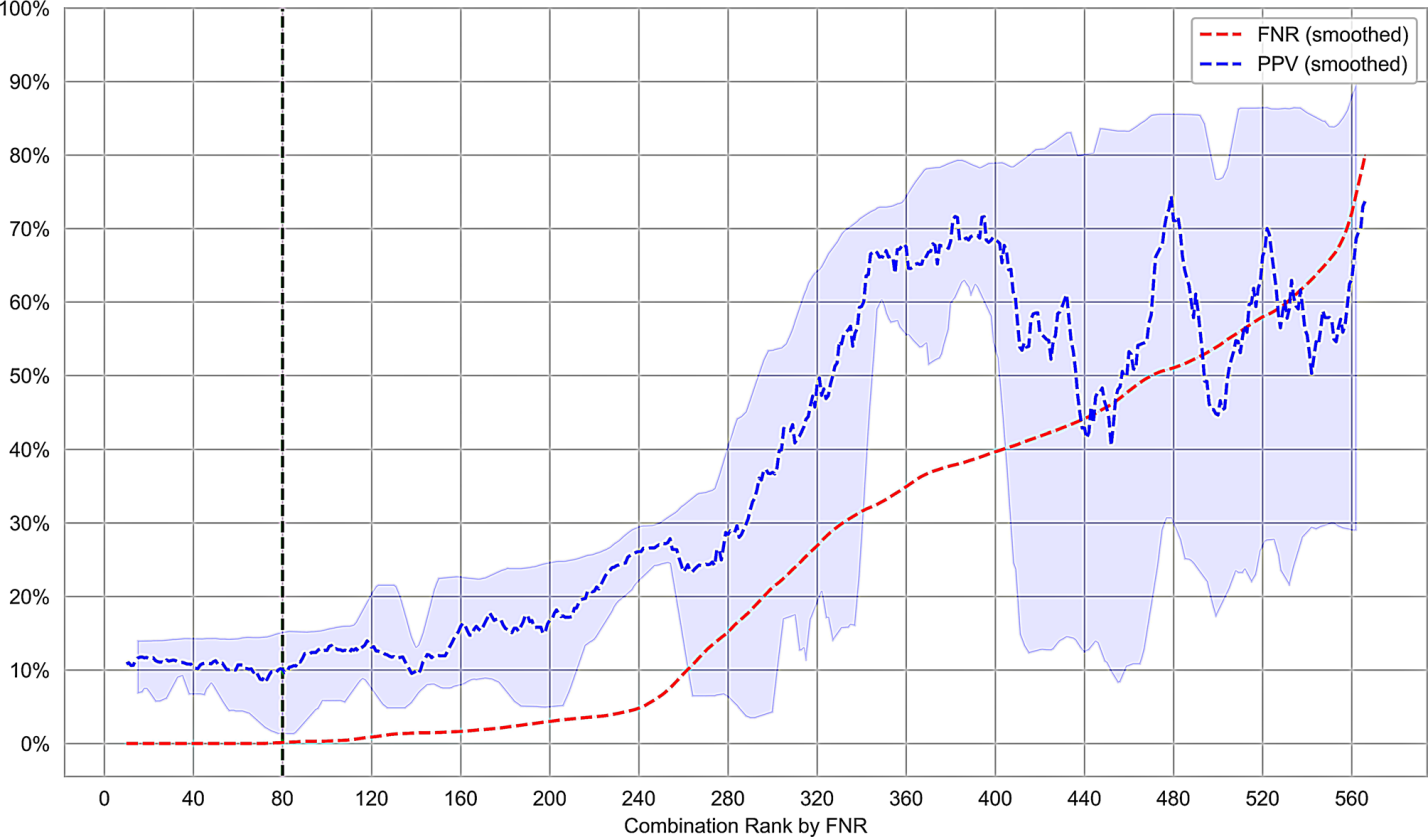
PPV and FNR of the search space, ranked by ascending FNR on the x-axis. Models ranked to the left of the green line have an FNR of 0.0% indicating they may be viable models. These models are shown in Fig. 4

The overall relationship between FNR and PPV is shown in Fig. 5, with the more detailed Fig. 4 showing all models achieving a 0% FNR. The best performing models are those that achieve a 0% False Negative Rate (FNR), shown to the left of the green dash line, sorted by PPV. There is significant variability in model PPV across varying FNR levels. The shaded blue region represents the smoothed 10th and 90th percentile of PPV across the 30 models making up the smoothed dashed blue line.

### Explainability

**Fig. 6:**
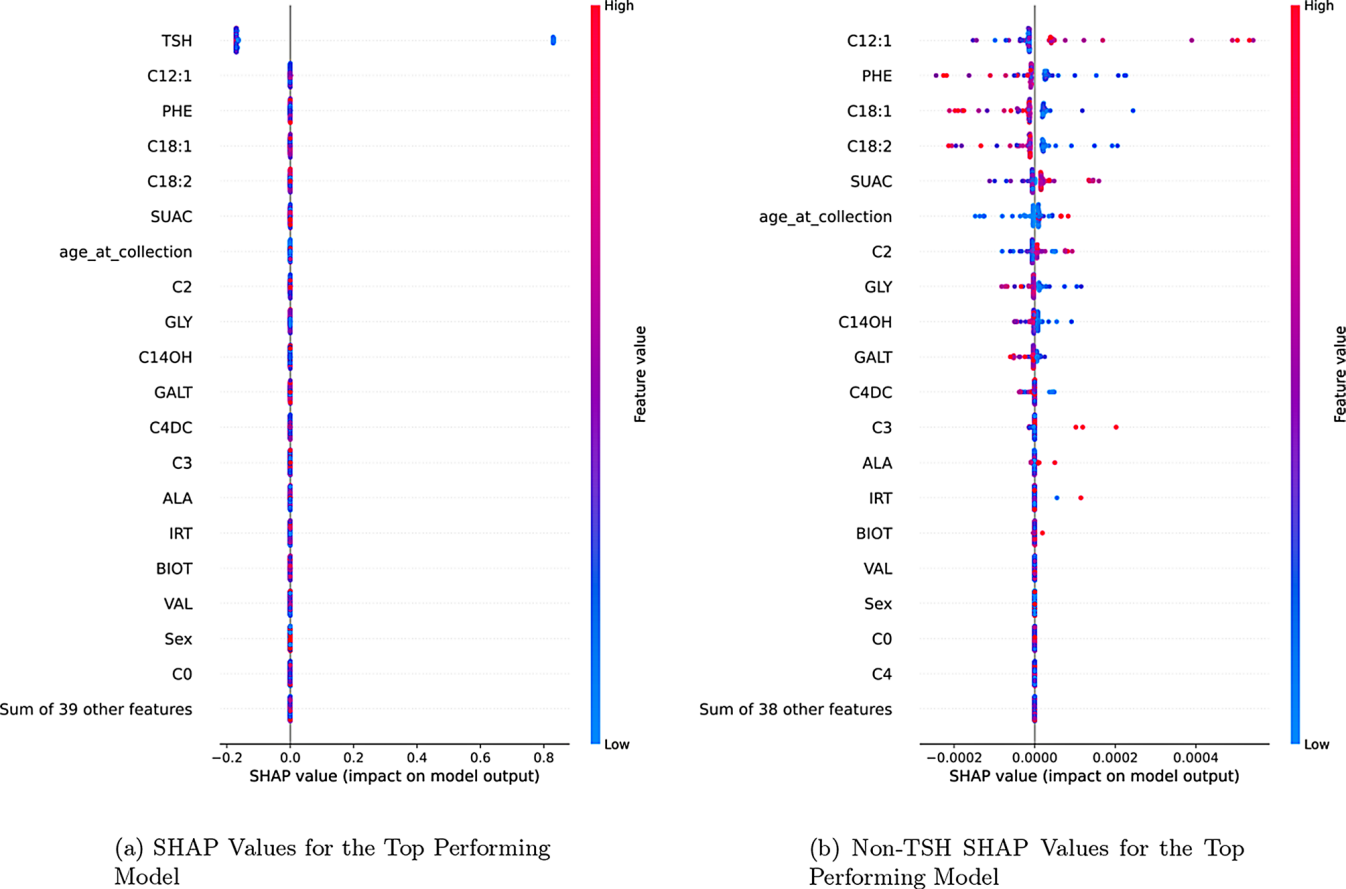
Comparison of shap value distributions with (left) and without (right) TSH feature inclusion

**Fig. 7:**
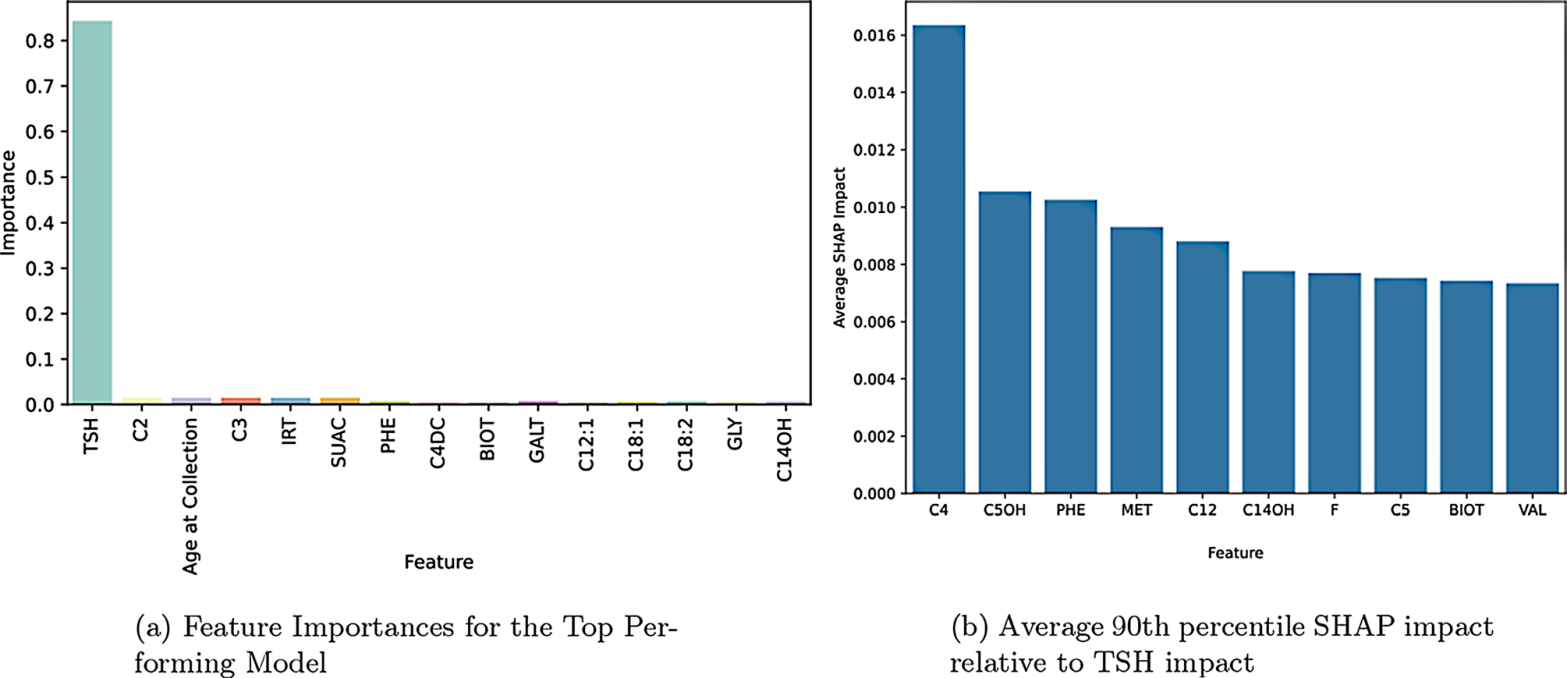
Combined visualization of model interpretability metrics

The top performing models were evaluated using SHAP and feature importances. The results of the SHAP values for the top performing model are shown in Fig. 6a. The dominance of TSH in the plot may appear as if there is no impact from other features. Figure 6b shows the very minor, but existent, impact of other features on the output of the model. The feature importances for the top performing model are shown in Fig. 7a. The results of the SHAP values and feature importances show, as expected, that TSH is the most important feature in the model by far, but that there is at least some minimal impact from the other features.Fig. 6Comparison of shap value distributions with (left) and without (right) TSH feature inclusionFig. 7Combined visualization of model interpretability metrics

In the other models that were evaluated, the SHAP values and feature importances show a similar pattern. There is however in some cases variability in the importance of the other features, as indicated by the feature importances and SHAP values. The 90th percentile of SHAP impact values across each of the top ten models were collected. The SHAP impact values relative to the impact of TSH is shown in Fig. 7b.

**Fig. 8:**
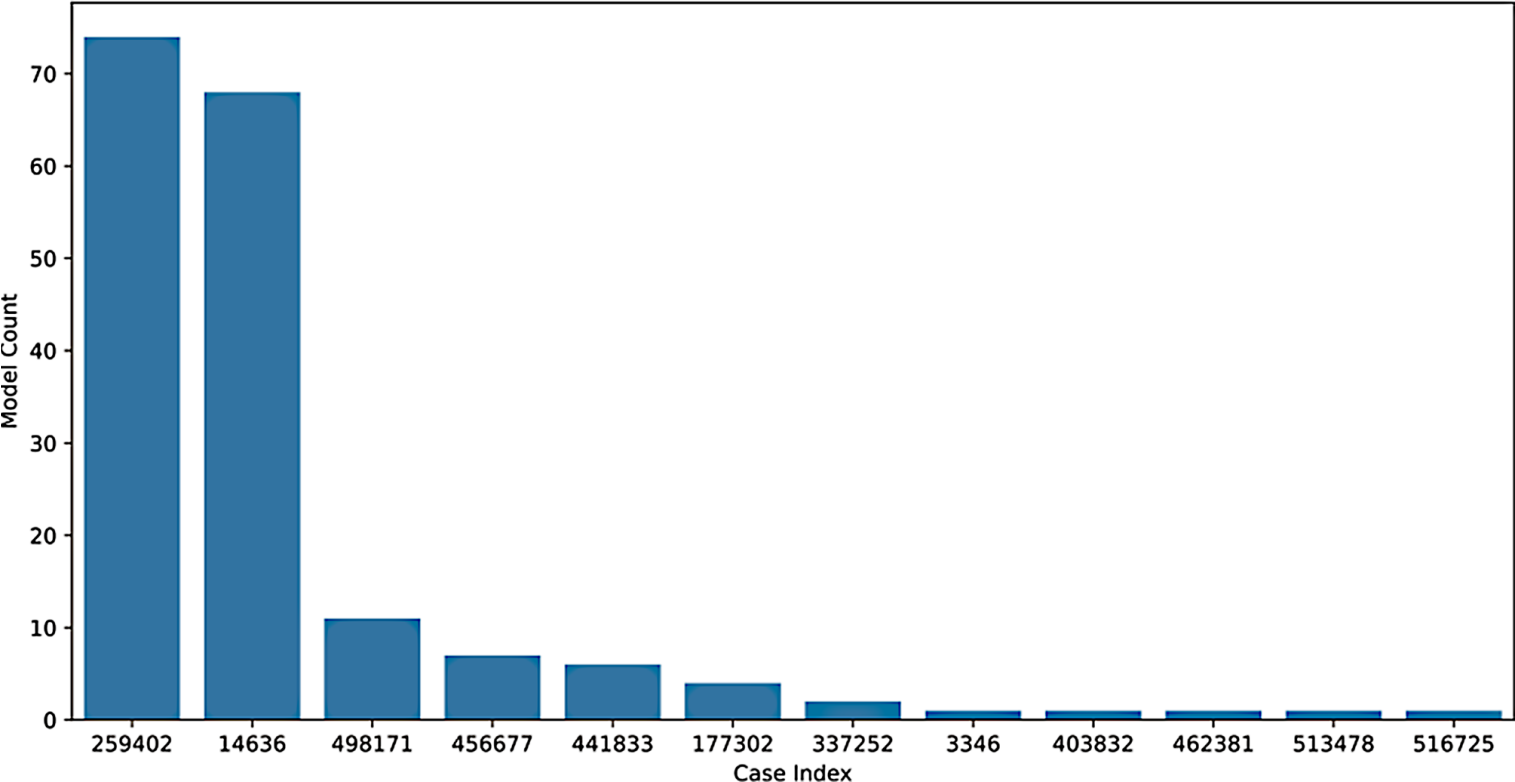
Frequency of misclassification of the most difficult true positive cases of CH across the search space

There were two true positive cases in particular that proved challenging for the models to predict. Figure 8 shows these two points as outliers across the search space (cases 259,402 and 14,636). One should note however that this represents approximately 12% FNR for these two points over the 576  classification attempts in the search space.

**Fig. 9:**
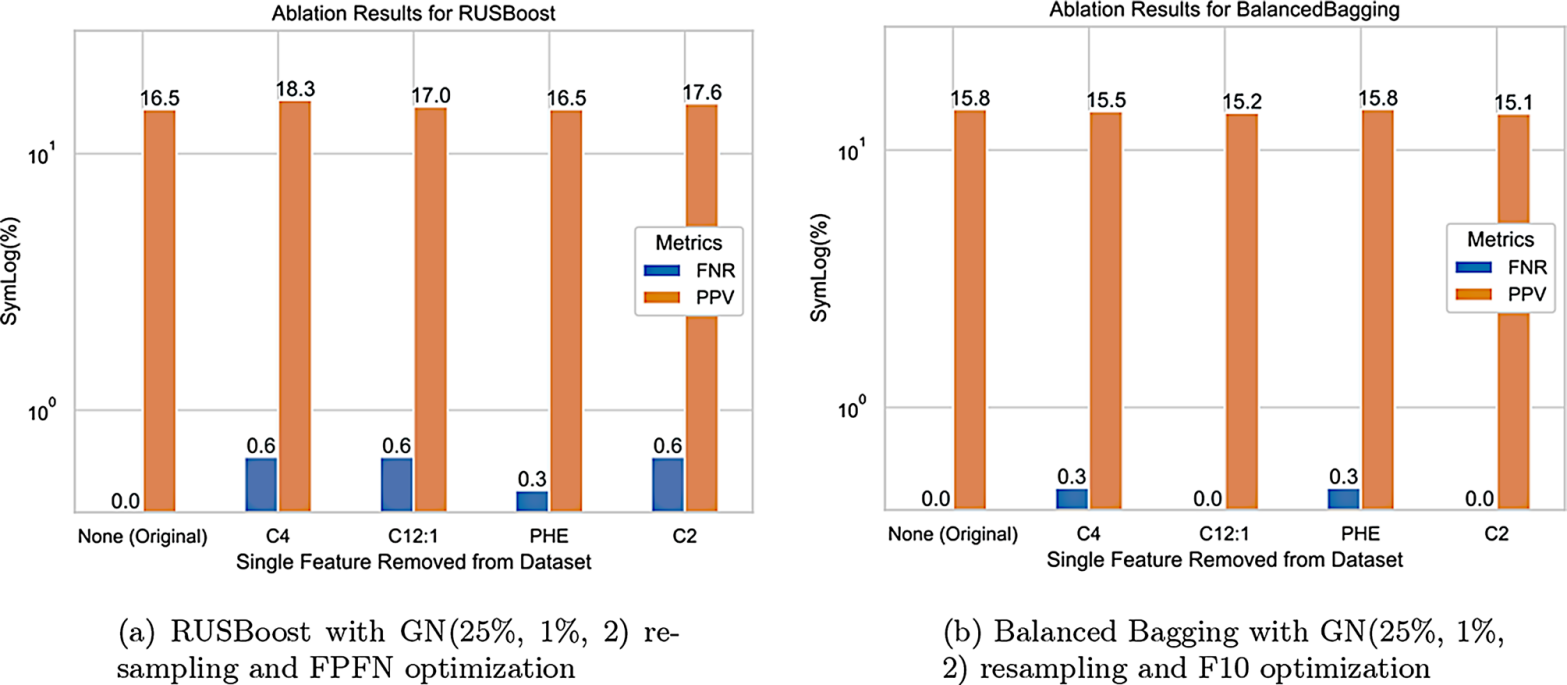
Ablation study results on the two top performing models: performance after removing a single feature of relatively high importance, as determined by shap value and feature importance

We also perform a small ablation study for the top two performing models; removing the top 5 most impactful features individually. These were selected via both SHAP values (C12:1, PHE, C4) from Figure 6b, feature importance (C2) from the top performing model (Fig. 7a), and average SHAP impact relative to TSH shown in Figure 6b. The results of this feature removal are shown in Figure 9, and show either hindered PPV or an increased FNR. This confirms that there is an impact of these secondary features on model performance, but that is not consistent in magnitude across different models.

### Combinations of models

Unanimous voting of the best performing models increased performance from 16.8% to 18.2% PPV. The results of these combinations are summarized in Table 8. These improvements suffer from diminishing returns as the number of models in the ensemble increases. with negligible improvements after including 3 models in the system.

**Table 8:** Models used in the ensemble voting system resulting in the best PPV (18.23%) with no false negatives

Classification	Resampling	Scoring	FNR %	PPV %
RUSBoost	GN (25%, 1%, 2)	FPFN	0.00	16.85
Balanced Bagging	GN (25%, 1%, 2)	F10	0.00	15.80
Balanced Bagging	GN (25%, 5%, 2)	F10	0.00	14.70
RUSBoost	Tomek Links	F10	0.00	14.42
Balanced Random Forest	GN (25%, 1%, 2)	FPFN	0.00	14.31
Balanced Bagging	GN (25%, 1%, 2)	F1	0.00	14.28
**Combined**			**0.00**	**18.23**

We also show the overlap between the top ten models in Fig. 9. In this figure, each case predicted as positive by the top 10 models is a column. The top row shows the predicted positive probability of all cases. This probability is used to sort the cases. The bottom row contains the true positive cases marked in bright pink. The rows in between are the predicted positive cases by each model. In the cases where there is whitespace, this indicates that the model did not predict the case as positive. Models are sorted in ascending order of PPV from bottom to top.

**Fig. 10:**
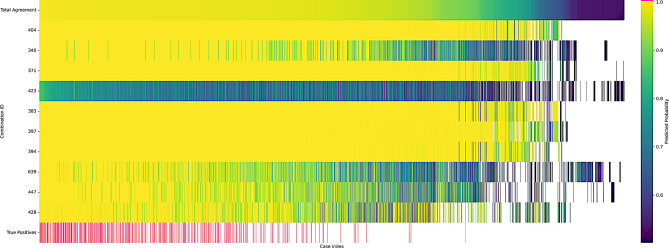
Individual positive predictions of the top-ten best performing models within the search space. Each column represents a case, where the color of a given row represents the predicted probability of the case being positive. White represents that a positive prediction was not made for that case. True positives are shown in the bottom row in pink

## Discussion

By improving the PPV of the CH screening process at NSO, we can reduce the number of infants who are unnecessarily retested, reducing tests and stress for families. The best performing model in this study achieved a PPV of 16.8%, representing a 60% improvement over the current first-tier screening process. This model was a RUSBoost model with a FPFN optimization metric and Gaussian Noise resampling, GN$\left(25\%, 1\%, 2\right)$. The SHAP values and feature importances of the top-performing model highlight the importance of TSH levels in CH prediction. While the additional features in the dataset show some impact on the model, TSH remains the primary predictor. This study shows promise for the use of machine learning globally in CH screening programs in a real-world setting, with multiple potential avenues for future advancement.

We would like to note that the current 10.5% PPV of the existing NSO CH screening process reflects the logic described in Section 1.2 and was not evaluated using the 5-fold cross validation method as we did for all ML models. The performance of this process is evaluated separately on the same dataset by NSO [7].

Removing approximately 10% of our dataset from NSO due to missing or malformed data is a limitation of this study. The greatest removal of cases is due to DBS collection outside the 24 to 176-hour window, which is necessary to ensure the accuracy of the TSH measurements. TSH is expected to be high immediately after birth and decline rapidly in the 24 hours post-birth [29]. Cases that are missing metabolic data are not useful for training a model, as the model relies on metabolic features. These two issues are the cause of the majority of removed cases. Variations in the collection of DBS samples are also a limitation in current screening programs that can be overcome by retesting within the appropriate age window post birth. If TSH is available but not other metabolic features, use of the current threshold based program would be appropriate. Given the dominance of TSH in the model, it is not unlikely that substituting the missing values with a mean or median value would still produce useful predictions, however, this is not explored in this study.

The closest study to this, Zarin Mousavi et al. [11], used a very limited dataset that was not reflective of the general population, and failed to produce acceptable results. Machine learning has only been shown to improve CH screening when there are multiple dominant features, as in Jansen et al. [14] and Stroek et al. [13]. Similarly, our findings reveal that there is information present in the dataset beyond endocrine markers, in our case TSH. It is difficult to make a direct comparison between only using TSH and using all features available in the dataset, as training models only on TSH would lead to either overfitting or a threshold model. We are able to show some small impact of non-endocrine features, that are not known to correlate with CH, on the model. This is in keeping with the findings of Jansen et al. [14].

In order to gain a better understanding of the behaviour of the top models and non-endocrine features, we investigate the average maximum SHAPley interaction values for these features. These SHAPley interaction values provide a deeper understanding of how features interact within the model space on a case by case basis. Given the relatively low impact of these features, when they are used, what is the most impact they can have. By taking the 90th percentile of SHAPley values across the top 10 most performant models, we can show a different view of feature importance. The specific non-endocrine features that a given model selects to use as decision boundaries are not entirely consistent. This can be seen in the differences between Fig. 6b, 7b, and the additional SHAP plots in Appendix A. Given the variation among the top models, speculation as to the contribution of these specific features may be best left to further research.

Given the minimal impact of SHAPley values we opted to perform a small ablation study. In this we remove a single feature and evaluate the top two models without said feature in train or test sets. This had clear impacts on model performance, either decreasing sensitivity, introducing false negative screen results, which are unacceptable, or lowering the PPV which is not ideal. This is not unsurprising given the SHAPley values and feature importances. We did however attempt to shed some additional light on the impact of these variables through a secondary analysis of feature relationships. Using a bag plot, we do not find obvious visual indicators of why the four selected features impact the models enough to introduce false negative predictions. These plots are available in Appendix C.

Our best models contradict the approach taken by other studies which only investigate Random Forest models [11, 13, 14]. We show that a variety of classifiers can be effective in learning the data distribution of CH screening data, but the learner chosen has a significant impact on the performance of the model. The same can be said of resampling, existing studies only investigate SMOTE resampling if resampling is performed at all [11, 13, 14]. While SMOTE is shown to improve diagnostic performance in these studies, opposed to no resampling, we show that other resampling techniques can be potentially more effective in improving the performance of the model [13]. The optimal choice of resampling is clearly dependent on the dataset and model used, we show that despite the popularity of SMOTE, it is not always the best choice, or even near to the best choice. The optimization metric used in this study is also different from other studies, where F1 is typically used [11, 13, 14]. It is a common mistake for studies to use F1 optimization and retroactively adjust the decision threshold to achieve a desired FNR, this is not a valid approach as it is not generalizable to the general population. The choice between FPFN and F10 optimization is not clear, but we show that both can be effective in training a model that can achieve a 0% FNR and an improved PPV compared to the current screening process.

The unanimous voting of the best performing models, achieving a small increase to 18.23% PPV without any false negatives, cannot be said to generalize well to unseen data yet. The downside of improved PPV is that there are multiple sources of error from each of the constituent models. We have not evaluated the generalizability of the model to the general population, but the overlap between the models is a good indicator that the models are learning similar aspects of the data distribution. With this being known, the upside of the unanimous voting is not enough to warrant the increased risk of error or the increased complexity of the model. A more fully studied ensemble of different models may be interesting to investigate in future work but cannot be said to be a conclusive improvement in this study.

The most successful models were not exclusively those trained on Gaussian noise resampling, but 5 out of 6 of the models selected for unanimous voting used Gaussian noise resampling during training (Table 8). This may indicate that the resampling did not increase noise in the feature space to the point of harming performance but did increase the learnability of the dataset in slightly different ways.

The plot in Fig. 9 shows the lack of agreement in the less probable predictions of the models. It was thought that this was due to the models learning different aspects of the data, but the lack of improvement from the unanimous voting indicates that this is not the case. The models are learning similar aspects of the data, but are not able to agree on the less probable cases. This is likely due to the inherent variability in the data and the difficulty in learning from the two outlying cases with low TSH levels. There are two outlier cases that are very often predicted as negative by the unacceptable models, they appear in the rightmost third of Fig. 9. These two cases are clearly the most difficult to learn based on the frequency of misclassification compared to the other true positive cases as seen in Fig. 8. These two cases have a TSH concentration of 15.2 and 15.4 mIU/L, well below the other positive samples in the dataset, which are all above 16.0 mIU/L. These cases are likely the most difficult to learn from, as they are the only two cases in the dataset that are below 16.0 mIU/L. If these two cases are both in the test set of the stratified cross fold validation, the model will not be able to learn from any cases with TSH under 16.0 mIU/L and will thus most likely not be able to achieve a 0% FNR.

This is perhaps why Gaussian noise resampling performs highly. It is difficult to learn effectively from a single outlier training example, but by adding noise around this outlier, the model is able to learn from the noise and generalize well. This could likely also be said about Tomek Link and Near Miss resampling, which undersample the majority class in a way that is likely to improve the learnability of the outlier region. Alternative oversampling methods like SMOTE are more likely to introduce noise that is not representative of the data distribution, and thus are less likely to improve the learnability of the outlier region. To illustrate using a one dimensional example, if TSH was oversampled using SMOTE, we would likely oversample into the region $15.3 \le 16.0$mIU/L, this would introduce noise that is not representative of the data distribution and would likely not improve the learnability of the outlier region. This may only pose an issue in our dataset, but this is likely a common issue in imbalanced datasets more generally. More examples would be necessary to fully evaluate this hypothesis, but it is a potential avenue for future work. Optimizing the Gaussian noise hyperparameters may also be a fruitful area for future work, as the hyperparameters used in this study were chosen arbitrarily.

Typically, machine learning algorithms will struggle to learn effectively when there is a single dominant feature. From both the feature importances and SHAP plots of different models, TSH is clearly the dominant feature. This can lead to issues for Random Forest based and similar algorithms that only test a subset of features at any given split, which in this case likely leads to poor branching structure. Models are obviously able to learn from the other features in the dataset, as the Linear SVM models are not able to achieve performance nearing the top 10 models. If there is no information in the other features, the SVM would effectively form a threshold on TSH levels according to the optimization metric. We show robustness against overfitting by using stratified cross fold validation, this is a common technique in machine learning to ensure that the model is generalizing well to the data [22].

In another potential avenue for future work, cost sensitive learning could be used to improve the performance of the model. This is a technique that is used to adjust the cost of misclassification based on the class of the sample. This is a common technique in imbalanced learning and could be used to improve the performance of the model. This is not used in this study as it is not a common technique in machine learning and is not widely understood [22]. We perform something similar when we integrate different optimization metrics into the model, but this is not the same as cost sensitive learning. Cost sensitive learning is a more flexible approach that can be used to adjust the cost of misclassification based on the class of the sample. This is a common technique in imbalanced learning and could be used to improve the performance of the model.

## Conclusion

This study evaluated the potential of 12 ML classification algorithms, 12 resampling algorithms, and 4 optimization metrics to improve the PPV of CH screening while maintaining high sensitivity. By leveraging a large dataset of 616,910 newborn screening samples from NSO, we were able to assess model performance in a more realistic scenario than previous studies.

Our findings demonstrate that machine learning can significantly improve the performance of TSH threshold-based CH screening, while maintaining 100% sensitivity relative to the existing program. Ensemble methods like RUSBoost, and Bagging algorithms, combined with Gaussian Noise resampling, showed the most promising results in handling the extreme class imbalance inherent in CH & rare disease screening. The inclusion of additional metabolic markers beyond TSH, such as amino acids and acyl carnitines, contributed to improved performance, suggesting these features contain information relevant to CH prediction. Careful selection of evaluation metrics and optimization strategies proved crucial when dealing with highly imbalanced datasets. Metrics like F10 score that emphasize recall were more suitable than traditional measures like accuracy or even F1 score.

By leveraging large-scale screening data and class imbalance specific solutions, we can develop more sensitive and efficient screening tools that benefit both patients and healthcare providers. With new understanding of the data and algorithms behavior, we can further improve the performance of the model and potentially reduce the number of false positives in the screening process.

## Electronic supplementary material

Below is the link to the electronic supplementary material.

Supplementary Material 1

## Data Availability

The data that support the findings of this study were provided by NSO and CHEO. Restrictions apply to the availability of these data, which contain sensitive personal information, and so are not publicly available. Researchers can request the use of newborn screening data through the Institute for Clinical Evaluative Sciences, where newborn screening data is available along with other clinical and administrative data sets. Newborn Screening Ontario may also receive requests for newborn screening data from the scientific community via our Medical Scientific staff. All studies using NSO data must have Research Ethics Board approval.

## Abbreviations

AA: Amino acids
AC: Acyl carnitiness
ADASYN: Adaptive Synthetic Sampling
AUC: Area Under the Curve
CH: Congenital hypothyroidism
CHAID: Chi-squared Automatic Interaction Detection
CHEO: Children’s Hospital of Eastern Ontario
DBS: Dried Blood Spots
FNR: False Negative Rate
GN: Gaussian Noise
ID3: Iterative Dichotomiser 3
ML: Machine Learning
MLP: Multilayer Perceptron
NBS: Newborn Screening
NSO: Newborn Screening Ontario
PPV: Positive Predictive Value
REB: Research Ethics Board
ROC: Receiver Operating Characteristic
SHAP: SHapley Additive exPlanations
SMOTE: Synthetic Minority Oversampling Technique
SVC: Support Vector Classifier
SVM: Support Vector Machine
T4: Thyroxine
TBG: Thyroxine-binding globulin
TSH: Thyroid-stimulating hormone
