# Supplementary material for: Enhancing TSH-based congenital hypothyroidism screening using machine learning and resampling algorithms
Source: BMC Med Inform Decis Mak. 2025 Dec 22;25:449. doi: 10.1186/s12911-025-03312-0 (PMC12751215; doi:10.1186/s12911-025-03312-0)
Supplement: Supplementary file 1 — Supplementary Material 1 [file 12911_2025_3312_MOESM1_ESM.pdf]

Appendix A Data Preprocessing & Summary Statistics

Figure A1 shows the preprocessing pipeline used in this study. The data comes from two sources, screening data and diagnostic data of screen positive cases. Figure A2 shows the number of malformed or missing values for each feature. These are each reason to remove a sample from the unprocessed dataset. There are clear groupings where the features are missing across many cases as they are measured together; for example the simple metabolic features, if one is missing, all will be missing.

The tables in this section show summary statistics of the preprocessed data. Table A1 shows detailed statistics and range of each feature included in the dataset. Table A2 shows a summary of the mean values for each feature, separated by definitive diagnosis and sex.

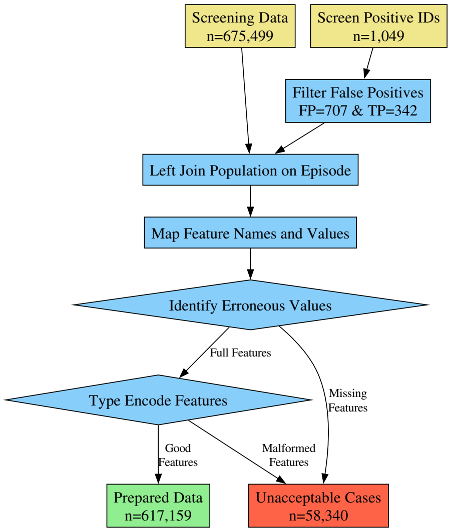

Figure A1: Preprocessing Flowchart for NSO Data

Feature	Mean	STD	Min	25%	50%	75%	Max
Gestational age	272.77	12.88	141.0	268.0	274.0	280.0	344.0
Birth Weight	3299.42	574.45	36.0	2990.0	3330.0	3660.0	34250.0
Age at Collection	27.54	9.89	24.02	24.28	24.72	26.37	167.97
ALA	220.08	64.72	42.0	176.0	210.0	252.0	3986.0
ARG	13.98	10.29	0.0	9.0	12.0	17.0	3672.0
CIT	16.20	5.26	1.0	13.0	16.0	18.0	1206.0
GLY	484.22	110.99	127.0	411.0	471.0	541.0	17534.0
LEU	105.06	25.95	26.0	89.0	102.0	117.0	5559.0
MET	26.88	7.10	3.0	22.0	26.0	30.0	663.0
ORN	80.48	29.37	0.0	61.0	75.0	93.0	1141.0
PHE	61.05	12.66	14.0	53.0	60.0	67.0	1591.0
SUAC	0.77	0.43	0.0	0.52	0.79	1.02	50.36
TYR	86.97	31.91	5.0	66.0	81.0	102.0	788.0
VAL	119.48	29.92	36.0	100.0	115.0	133.0	3921.0
C0	34.87	12.99	3.5	25.9	32.5	41.0	210.2
C2	29.35	10.06	3.1	22.4	27.6	34.3	274.0
C3	2.15	0.92	0.04	1.51	1.97	2.57	22.38
C3DC	0.09	0.03	0.0	0.07	0.08	0.1	1.98
C4	0.28	0.17	0.0	0.19	0.25	0.32	69.19
C4DC	0.61	0.22	0.06	0.45	0.57	0.72	5.8
C4OH	0.48	0.22	0.0	0.33	0.44	0.59	4.04
C5	0.13	0.06	0.0	0.09	0.12	0.15	9.71
C5:1	0.02	0.01	0.0	0.02	0.02	0.03	0.63
C5OH	0.14	0.08	0.0	0.11	0.13	0.17	15.11
C5DC	0.09	0.03	0.0	0.07	0.09	0.11	7.83
C6	0.07	0.03	0.0	0.05	0.07	0.09	4.14
C6DC	0.10	0.04	0.0	0.07	0.1	0.12	2.93
C8	0.09	0.16	0.0	0.07	0.09	0.11	49.25
C8:1	0.33	0.17	0.0	0.22	0.3	0.41	2.9
C10	0.11	0.05	0.0	0.08	0.1	0.13	3.14
C10:1	0.11	0.05	0.0	0.07	0.1	0.13	3.7
C12	0.17	0.07	0.0	0.13	0.16	0.21	3.45
C12:1	0.08	0.05	0.0	0.05	0.08	0.11	0.68
C14	0.26	0.09	0.03	0.2	0.25	0.3	35.42
C14:1	0.16	0.07	0.0	0.11	0.15	0.2	4.62
C14:2	0.05	0.02	0.0	0.03	0.04	0.06	0.85
C14OH	0.04	0.02	0.0	0.03	0.04	0.05	0.37
C16	2.92	0.92	0.16	2.27	2.80	3.44	24.67
C16OH	0.04	0.02	0.00	0.02	0.03	0.04	1.13
C16:1OH	0.08	0.03	0.00	0.06	0.07	0.10	3.69
C18	0.84	0.28	0.06	0.65	0.80	0.99	17.41
C18:1	1.29	0.38	0.05	1.02	1.25	1.51	11.28
C18:2	0.15	0.08	0.00	0.10	0.13	0.18	5.87
C18OH	0.02	0.01	0.00	0.01	0.01	0.02	0.58
C18:1OH	0.02	0.01	0.00	0.02	0.02	0.03	0.72
BIOT	127.47	31.26	0.00	105.80	125.90	147.40	334.90
GALT	8.61	2.25	0.40	7.00	8.50	10.00	22.70
IRT	23.46	13.45	1.50	15.20	20.60	28.30	1508.10
TREC_QN	573.05	317.97	0.00	356.00	514.00	717.00	6612.00
TSH	5.05	5.02	0.00	3.30	4.60	6.20	568.90
HGB_Pattern	0.97	0.16	0.00	1.00	1.00	1.00	1.00
A	15.55	5.86	0.20	11.40	14.80	18.80	86.20
F	58.83	5.44	1.20	55.60	59.30	62.70	78.70
F1	20.44	3.04	1.60	18.30	20.20	22.50	50.90
FAST	3.50	1.01	0.50	3.00	3.40	3.80	54.30

Table A1: Feature Detail Statistics

Diagnosis	Positive		Negative	
Sex	Female	Male	Female	Male
N =	196	146	300668	315807
gestational_age	272.9643	275.0685	272.7685	272.768
birth_weight	3182.6633	3377.8699	3299.4594	3299.4038
age_at_collection	26.9624	26.1784	27.5431	27.5432
transfusion_status	0.0102	0.0068	0.0033	0.0033
multiple_birth_rank	0.051	0.0137	0.0323	0.0323
ALA	238.6582	238.726	220.079	220.0805
ARG	13.7347	14.9795	13.9818	13.9815
CIT	17.4184	17.5753	16.2024	16.2025
GLY	509.9286	483.9247	484.2134	484.2217
LEU	103.801	105.1781	105.0619	105.0614
MET	28.9184	30.0685	26.8842	26.8841
ORN	84.9949	82.0411	80.474	80.4751
PHE	65.1837	67.7123	61.0481	61.0478
SUAC	0.7919	0.7445	0.7745	0.7745
TYR	90.1224	84.726	86.971	86.9726
VAL	118.9949	117.6644	119.4759	119.4762
C0	34.3092	36.7733	34.8727	34.8721
C2	26.6179	29.163	29.3547	29.3539
C3	2.0033	2.1355	2.1479	2.1479
C3DC	0.086	0.0923	0.086	0.086
C4	0.3386	0.2958	0.2786	0.2786
C4DC	0.5514	0.6128	0.609	0.6089
C4OH	0.4455	0.4673	0.4828	0.4828
C5	0.1591	0.1297	0.1279	0.1279
C5:1	0.025	0.0219	0.0219	0.0219
C5OH	0.1384	0.1405	0.1442	0.1442
C5DC	0.0951	0.1011	0.0912	0.0912
C6	0.0762	0.0763	0.0734	0.0734
C6DC	0.0945	0.0969	0.1013	0.1013
C8	0.0887	0.0996	0.0925	0.0925
C8:1	0.3444	0.3318	0.3328	0.3328
C10	0.1004	0.1142	0.1086	0.1086
C10:1	0.1029	0.1054	0.1052	0.1052
C12	0.1597	0.1784	0.1747	0.1747
C12:1	0.0812	0.0888	0.0843	0.0843
C14	0.2513	0.2755	0.2586	0.2586
C14:1	0.156	0.1696	0.1627	0.1627
C14:2	0.0439	0.0468	0.0461	0.0461
C14OH	0.0444	0.0449	0.0443	0.0443
C16	2.7194	2.9022	2.9172	2.9171
C16OH	0.0346	0.037	0.0359	0.0359
C16:1OH	0.0734	0.0807	0.0789	0.0789
C18	0.8121	0.806	0.8422	0.8422
C18:1	1.225	1.2655	1.2925	1.2925
C18:2	0.1427	0.1565	0.1502	0.1502
C18OH	0.0151	0.0143	0.0154	0.0154
C18:1OH	0.0214	0.0216	0.0219	0.0219
BIOT	126.5704	121.3596	127.4747	127.4759
GALT	8.1872	8.1904	8.6082	8.6081
IRT	23.2168	21.9959	23.4587	23.459
TREC_QN	546.852	427.089	573.0537	573.08
TSH	138.6837	113.7534	5.0027	5.0195
HGB_Pattern	0.949	0.9863	0.9746	0.9746
A	16.0342	15.6438	15.5491	15.5492
F	58.1092	59.1301	58.8322	58.8319
F1	20.4587	20.2705	20.4388	20.4389
FAST	3.6888	3.4445	3.4994	3.4995

Table A2: Feature Summary Statistics By Sex and Diagnosis

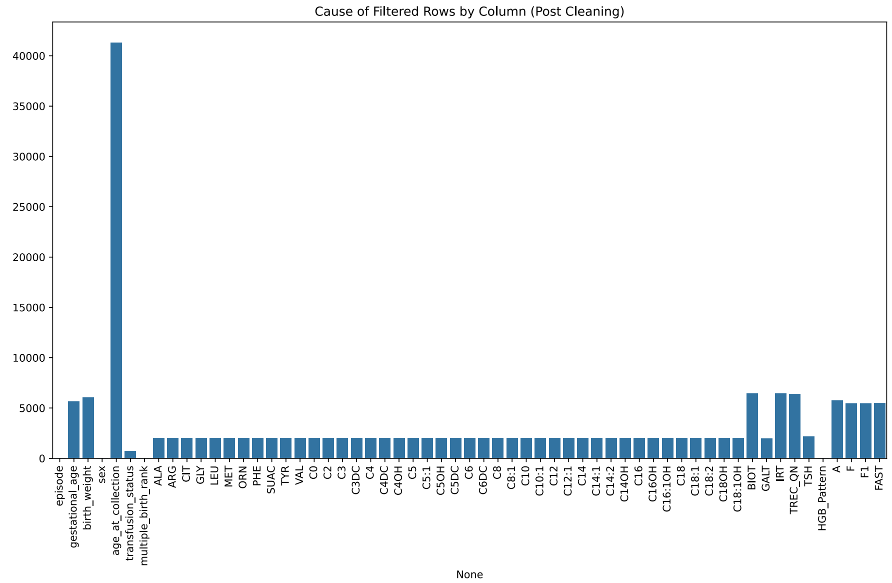

Figure A2: Cases containing irreparable values causing removal of a case

Appendix B Search Space Evaluation

The search space evaluation process is outlined in Figures Figure B3, Figure B4, Figure B5 in order of increasing detail.

The process begins by defining a collection of classifiers, resamplers, optimization metrics, and cross validation strategies as the search space. Our runtime engine takes as input the search space and the preprocessed data and runs each combination of algorithms. The results are then analyzed, and common evaluation metrics are calculated and stored automatically in a results' database. The results are then manually analyzed to determine the best performing model. This is shown in Figure B3.

For each combination of algorithms, the runtime engine creates a job and dispatches it to a worker node. The worker node then trains the model using the given combination of algorithms and the preprocessed data. The results are then stored in the results' database. This process is shown in Figure B4.

At the job level, the runtime engine provides the preprocessed data, which is then split into a training and testing set. The train set is then resampled if an algorithm is provided. With this resulting dataset, a model is trained using a given ML algorithm with Bayesian hyperparameter optimization and the provided optimization function. The resulting model is tested using the test set. The results are then stored in the results' database. This occurs as many times as defined by the given cross fold validation strategy, with analysis performed independently at each fold. This process is shown in Figure B5.

All Python code used in this study, performing the process outlined above, is available on the Newborn Screening Ontario GitHub [24]. The data however is not available due to privacy concerns.

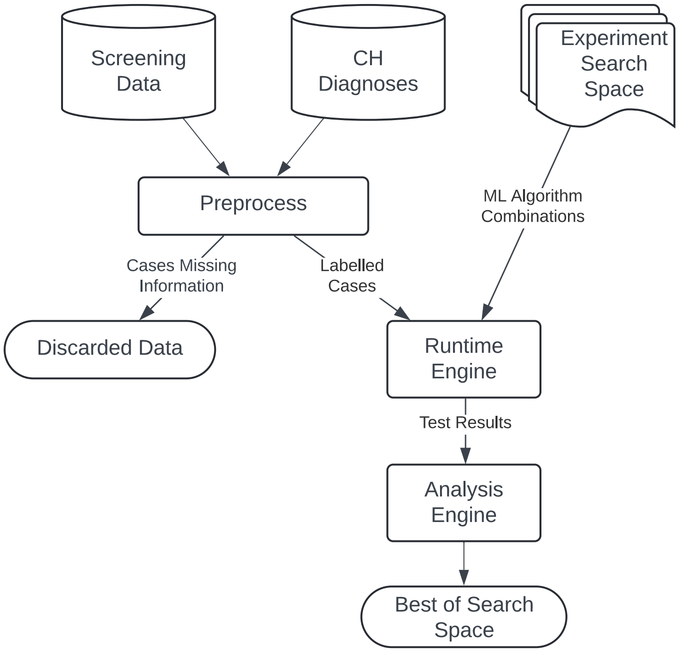

Figure B3: Overall Search Space Evaluation Process

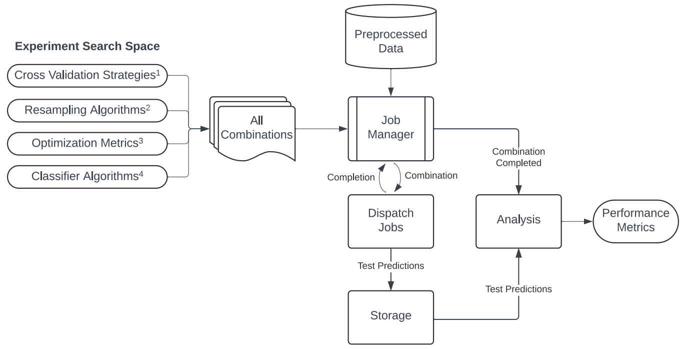

Figure B4: Job Dispatch Process

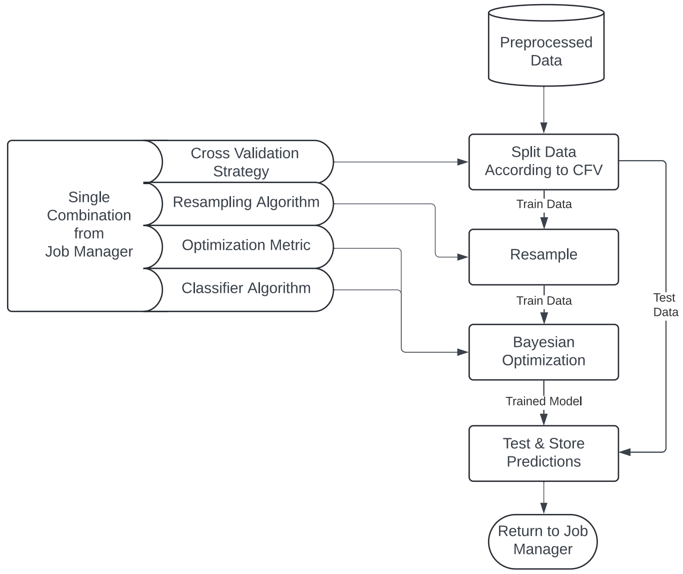

Figure B5: Job Detail Process

Appendix C Explainability

Below are several SHAP plots designed to provide insights into how machine learning models make predictions. These plots are based on SHAP values, which quantify the

contribution of each feature to a model's predictions. These plots also include a view in which TSH is removed, in order to be able to see the relative importance of secondary features to model predictions, and that they are in fact being drawn upon to inform predictions.

We also analyzed the relationship between TSH, Definitive Diagnosis of CH, and selected secondary variables (C12:1, PHE, C4, C2) as selected by impact on SHAP and feature importances of the top model. We hope this gives some better understanding of the slight impact of these variables on model predictions and why they are not substantially more predictive than TSH alone. Figure C7 show these relationships through the use of bag plots, which consist of a scatter plot and two box plots, all labelled by the ground truth labels. These plots may be analyzed visually to gain some better understanding of the relationships between these key features, although it is our belief that the impact is too small and likely too complex to derive significant understanding from these plots. They do, however, inform just how predictive TSH is compared to all other features in a visual manner.

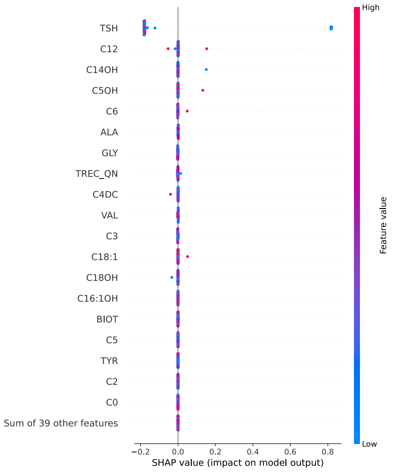

(a) SHAP values for the Balanced Bagging model trained using F10 optimization and GN(25%, 1%, 2) resampling.

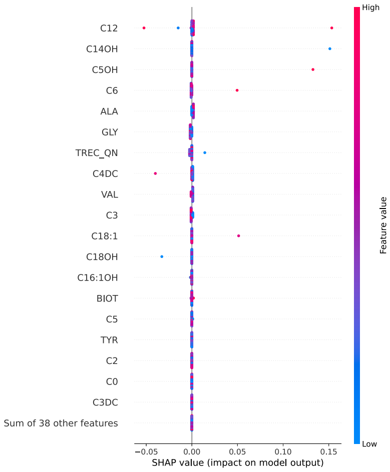

(b) SHAP values for the Balanced Bagging model trained using F10 optimization and GN(25%, 1%, 2) resampling without TSH values.

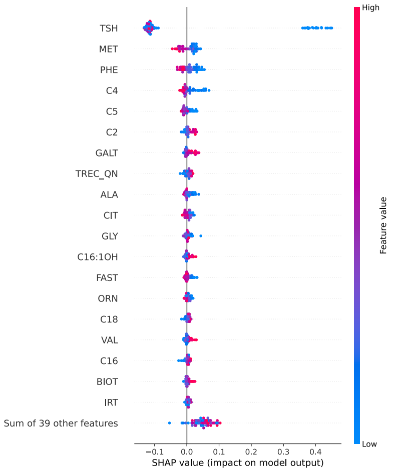

(c) SHAP values for the Balanced Random Forest model trained using FPFN optimization and GN(25%, 1%, 2) resampling.

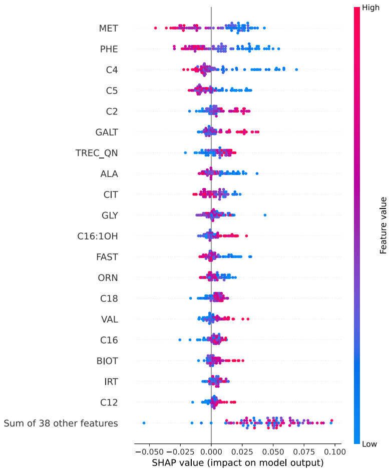

(d) SHAP values for the Balanced Random Forest model trained using FPFN optimization and GN(25%, 1%, 2) resampling without TSH values.

Figure C6: SHAP values for different models and resampling strategies.

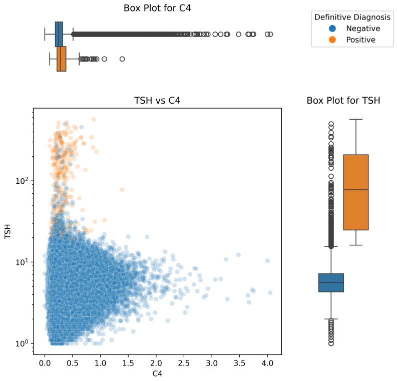

(a) TSH vs. C4

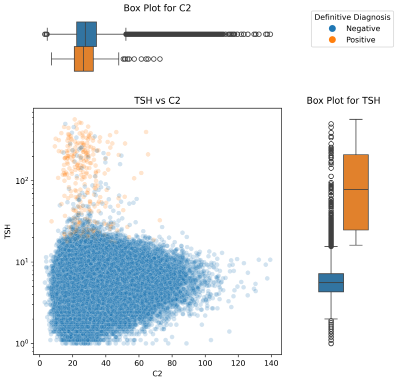

(b) TSH vs. C2

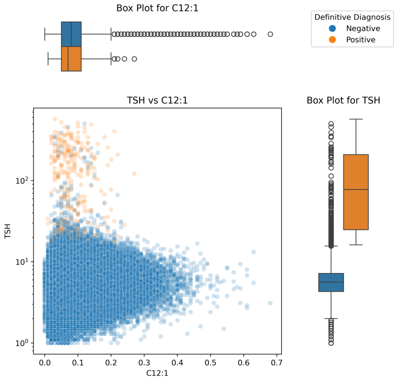

(c) TSH vs. C12:1

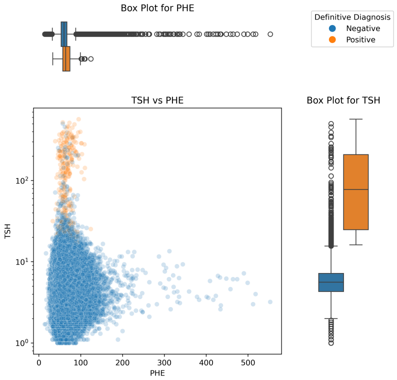

(d) TSH vs. PHE

Figure C7: Bag plot of the relationship between TSH,

Appendix D Gaussian Noise Algorithm

Algorithm 1 Gaussian Noise Resampling Implementation

Require:

X Data matrix
Y Labels of X
$r\%$ Features to apply noise to per sample
$m \in \mathbb{R}^+$ Noise multiplier of a feature σ
$p \in \mathbb{R}^+$ Ratio of synthetic to original samples

Ensure:

X' Data matrix with resampled points
Y' Labels of X'

procedure $(X,\ Y,\ r,\ m,\ p)$
  $F \leftarrow$ numeric feature indices from X
  $X_M \leftarrow$ majority class points in X
  $\forall f \in F : \sigma_f \leftarrow \text{std}(X_M[f])$
  $l \leftarrow \text{len}(X_M)$
  $X_r \leftarrow [0] \times l$
  ▷ Initialize new sample counts
  for $n \in \{0 \ldots \lceil p \cdot l \rceil - 1\}$ **do**
    $j \leftarrow \mathcal{U}(0, l-1)$
    $X_r[j] \leftarrow X_r[j] + 1$
  ▷ Apply noise to selected positive samples
  for $i \in X_r$ **do**
    $F' \leftarrow$ random subset of F of size len(F)
    for $f \in F'$ **do**
      $X_i[f] \leftarrow \mathcal{N}(X_i[f], \sigma_f \cdot m)$
  return $X \cup X_M, Y \cup Y_M$
